# More sensitive identification of psychotic experiences in common mental disorder by primary mental healthcare services – effect on prevalence and recovery: casting the net wider

**DOI:** 10.1192/bjo.2020.120

**Published:** 2020-11-06

**Authors:** Clare Knight, Debra Russo, Jan Stochl, Peter B. Jones, Jesus Perez

**Affiliations:** Department of Psychiatry, University of Cambridge, UK; Department of Psychiatry, University of Cambridge, UK; Department of Psychiatry, University of Cambridge; and NIHR Applied Research Collaboration (ARC) East of England, Cambridge, UK; Department of Psychiatry, University of Cambridge; and NIHR Applied Research Collaboration (ARC) East of England, Cambridge, UK; Department of Psychiatry, University of Cambridge; NIHR Applied Research Collaboration (ARC) East of England, Cambridge; and Norwich Medical School, University of East Anglia, Norwich, UK

**Keywords:** Anxiety, at-risk mental state, common mental disorder, depression, psychotic experiences

## Abstract

**Background:**

Psychotic experiences may emerge in more severe cases of common mental disorders (CMD). Previous work identified that 30% of patients treated by mental health services in primary healthcare, specifically the Improving Access to Psychological Therapies (IAPT) programme in England, reported psychotic experiences, began treatment with more severe CMD and were less likely to reach recovery.

**Aims:**

To replicate our previous assessment of psychotic experiences in the IAPT programme using a more sensitive threshold and determine its impact on the prevalence of psychotic experience and likelihood of recovery. Additionally, to compare recovery rates between patients with and without psychotic experiences at the end of therapy.

**Method:**

The Community Assessment of Psychic Experiences (CAPE-P15) with a cut-off of 1.30 was used to determine the prevalence of psychotic experiences. Recovery rates were determined using measures collected in the IAPT programme for depression (PHQ-9) and anxiety (GAD-7). Multi-group growth models estimated improvement trajectories.

**Results:**

In total, 2042 patients with CMD completed the CAPE-P15. The mean age was 39.8. The prevalence of psychotic experiences was 18% higher when using a lower threshold. The recovery rate for patients with psychotic experiences was lower (36%) than for those without (64%). Despite sharing similar improvement trajectories, the higher initial severity of CMD among patients with psychotic experiences impeded likelihood of recovery.

**Conclusions:**

As psychotic experiences may be a marker of severity in CMD, the benefits of identifying these in IAPT populations may also apply to patients with milder experiences. Further investigation of the consequential demands on service provision and how this would affect clinical practice is recommended.

Studies modelling the co-occurrence of common mental disorders (CMD), such as anxiety and depression, and psychotic experiences have revealed that such experiences measure severity of a unitary, latent continuum of common mental distress^1^ and correlate with increased comorbidity, suicidality and poorer treatment outcomes.^2–8^ Psychotic experiences also predict propensity to seek treatment from mental health services for persistent mental ill health.^9^ However, stringent referral thresholds prevent these individuals accessing secondary mental healthcare services and they are therefore managed in primary mental healthcare settings. In England, primary mental healthcare for CMD is mainly delivered by the Improving Access to Psychological Therapies (IAPT) programme (www.england.nhs.uk/mental-health/adults/iapt/). The programme was established to increase the access to National Institute for Health and Care Excellence (NICE) recommended psychological treatments within the National Health Service (NHS) across England.

## The challenge in IAPT

Although commissioned to treat mild to moderate CMD, IAPT services serve a clinical population with increasingly complex and comorbid conditions.^10–13^ As psychotic experiences emerge in more severe cases of CMD,^1^ we hypothesised in recent work that a proportion of the IAPT population would experience psychotic phenomena and, given the lack of specific treatment protocols for addressing psychotic experiences, we also predicted that this group would have poorer treatment outcomes.^14^ Using a cut-off score of 1.47 on the 15-item Community Assessment of Psychic Experiences-Positive (CAPE-P15) scale,^15^ a score previously calibrated^16^ against the Comprehensive Assessment of At-Risk Mental States (CAARMS),^17^ which identifies individuals with at-risk mental states for psychosis, we identified psychotic experiences in 30% of the IAPT case-load. This group of patients began therapy with more severe depression and/or anxiety and were significantly less likely to reach recovery by the end of treatment.^14^ However, if we understand psychotic experiences, depression and anxiety symptoms as manifestations of a continuum of distress, arguably, a cut-off of 1.47 may not identify all individuals with concurrent CMD and psychotic experiences in IAPT services. It is possible that those experiencing intermittent psychotic experiences will still have greater CMD severity and poorer outcomes. Recently, we have shown that the sensitivity of the CAPE-P15 can be increased (e.g. using a threshold of 1.30) to offer a useful choice of cut-off values to identify more people with psychotic experiences who may otherwise remain undetected but may nonetheless not receive optimal treatment by the IAPT programme in England.^18^

## Aims

The current study explored this by replicating our earlier assessment of the prevalence and impact of psychotic experiences among patients treated in IAPT services^14^ using a more sensitive CAPE-P15 cut-off score of 1.30. Specifically, we sought to determine the increase in the proportion of the overall case-load with psychotic experiences and compare the likelihood of recovery by the end of treatment in IAPT services between patients with and without comorbid lower-threshold psychotic experiences.

## Method

### Setting

The IAPT programme, which has transformed treatment of CMD in England, began in 2008 with the purpose of substantially increasing access to evidence-based psychological therapies for depression and anxiety disorders. Widely deemed a success, the programme has continued to expand over time to provide treatments to people with long-term and medically unexplained symptoms, with NHS England committing to covering 1.9 million adults aged 17–65 years annually by 2023–2024.^19^ Cognitive–behavioural therapy (CBT) is the predominant approach adopted by these services, although a wider range of treatments is offered, including counselling, interpersonal therapy and eye movement desensitisation and reprocessing. Interventions are delivered via telephone, face to face and, increasingly, online therapy in community settings and can be accessed by people in England who are registered with a general practitioner. Typically, the number sessions offered varies between 8 and 20, depending on local commissioning guidelines, over 3–4 months.

### Measures

An integral part of the IAPT programme is rigorous performance measurement and programme evaluation, and it therefore stipulates a minimum data-set to record the clinical progress of each patient. Patient-reported outcome measures of depression and anxiety are obtained at baseline and at each subsequent treatment session, ensuring that every patient has a clinical end-point even if treatment is discontinued unexpectedly. The measures used include the Patient Health Questionnaire (PHQ-9)^20^ and the Generalised Anxiety Disorder questionnaire (GAD-7),^21^ and services store these data using one of two patient management systems, PCMIS (www.york.ac.uk/healthsciences/pc-mis) or iaptus (iaptus.co.uk). This information is used to establish recovery from depression and anxiety and, although several outcome indices are used, the most commonly cited is the IAPT recovery index.^22^ This states that a patient has entered recovery if they score above the clinical cut-off on the PHQ-9 and/or GAD-7 at outset of treatment and below the cut-off on both the PHQ-9 and GAD-7 post-treatment, i.e. below 10 and 8 respectively. Nationally, 51% of patients accessing IAPT recover,^23^ although outcomes vary significantly and are influenced by factors including treatment fidelity, dose–response effects and the complexity and severity of case-loads across services.^22–24^

Our participating IAPT services also collected the current CAPE-P15.^15^ The CAPE-P15 is a 15-item self-report measure of the frequency and associated distress of psychotic experiences. It provides a mean per-item score on two subscales (frequency of psychotic experiences and associated distress), with higher scores indicating a higher frequency of psychotic experiences and an increased level of associated distress.^25^ We used a cut-off of 1.30 for both subscales to help identify patients with fewer and less intense psychotic experiences than identified with the previously recommended cut-off of 1.47.^16^

### Sample

The analysis was conducted on patients receiving treatment from IAPT services in three mental health trusts in England: Cambridgeshire and Peterborough NHS Foundation Trust (CPFT), Norfolk and Suffolk NHS Foundation Trust (NSFT) and Sussex Partnership NHS Foundation Trust (SPFT). Each serves a mixed urban and rural population and they together represent a wide range of socioeconomic deprivation.

To replicate our previous analysis,^14^ data from all patients who received IAPT treatment between the commencement and end of CAPE-P15 collection (February to December 2018) were included. Regardless of when each patient completed the CAPE-P15, recovery data were obtained from their initial session up until their end-of-care date.

All patients who were suitable for treatment under the IAPT programme across the three participating mental health trusts were eligible to complete the CAPE-P15 questionnaire. In two trusts (CPFT and SPFT), the CAPE-P15 was offered to patients during a treatment session or as a homework task. The third trust (NSFT) used a digital portal to remotely collect routine data, links to which were automatically sent out to patients by the service. Patients were approached to complete a CAPE-P15 once during their course of treatment, at any time point deemed appropriate by the therapist. It was accompanied by a short explanation of the evaluation and instructions for completion. Patients were informed that completing the CAPE-P15 was voluntary.

This study was approved by and registered with the official NHS Quality Improvement Programmes of all participating NHS mental health trusts, with confirmation by the UK Health Research Authority (hra.nhs.uk). Data analysis followed the guidelines established by the UK Anonymisation Standard for Publishing Health and Social Care Data (digital.nhs.uk).

### Statistical analysis

We compared age, gender and ethnicity between patients who scored 1.30 and above on the CAPE-P15 (hereafter referred to as CAPE-positive), those who scored below 1.30 (CAPE-negative) and those who did not complete a CAPE-P15 (no CAPE). Continuous variables were compared using *t*-test and categorical variables using chi-squared test (χ^2^). A *P*-value of less than 0.05 represents a statistically significant difference.

Prevalence and recovery rates are presented separately for CAPE-positive and CAPE-negative individuals and includes patients who had at least two sessions with the IAPT service and at least one appointment after completing a CAPE-P15. To be included in calculations of recovery rates, we stipulated that patients should have been discharged from the service having had at least one appointment after completing a CAPE-P15. Recovery was determined using the recovery index.^22^

Multigroup growth modelling, with CAPE-positive and CAPE-negative individuals comprising the groups, was carried out to estimate improvement trajectories for each group. As essential information regarding appointment number was not available at one site (NSFT) a subsample of *n* = 1149 patients from CPFT and SPFT were used here. This analysis was done in Mplus version 8 for Windows.^26^

## Results

In total, 2042 CAPE-P15 questionnaires were collected from 28 852 patients receiving IAPT treatment during the data collection period; this was 7% of the case-load.

### Participant sociodemographic characteristics

Table 1 presents a comparison of age, gender and ethnicity for CAPE-positive versus CAPE-negative individuals. We also included comparisons with individuals who did not complete a CAPE-P15, to identify any potential bias as the sample was a proportion of the case-load.

**Table 1:** Comparison of age, gender and ethnicity for participants who did and did not complete a Community Assessment of Psychic Experiences (CAPE-P15) questionnaire

	CAPE-P15 status, *n* (%)		
	Positive (*n* = 982)	Negative (*n* = 1060)	No CAPE (*n* = 26 810)
Age, years			
17	63 (6.4)	30 (2.8)	724 (2.7)
18–35	469 (47.8)	411 (38.8)	12 413 (46.3)
36–64	433 (44.1)	538 (50.8)	11 904 (44.4)
65+	2 (1.7)	81 (7.6)	1769 (6.6)
Gender			
Male	287 (29.2)	345 (32.5)	8633 (32.2)
Female	695 (70.8)	716 (67.5)	18 177 (67.8)
Ethnicity			
Asian/Asian British	2 (1.8)	15 (1.4)	375 (1.4)
Black/African/Caribbean/Black British	1 (0.8)	8 (0.8)	188 (0.7)
Mixed/Multiple ethnic groups	2 (1.8)	8 (0.7)	375 (1.4)
Mixed other	1 (0.8)	2 (0.2)	107 (0.4)
Not stated/not known	86 (8.8)	68 (6.4)	1662 (6.2)
Other ethnic group	8 (0.8)	3 (0.3)	161 (0.6)
White	837 (85.2)	956 (90.2)	23, 968 (89.4)

Positive, scored ≥1.30 on the CAPE-P15; negative, scored <1.30 on the CAPE-P15; No CAPE, did not complete a CAPE-P15.

### Age

The mean age of CAPE-positive individuals was 36.9 years (s.d. = 14.34) and it was 42.5 years (s.d. = 15.75) for CAPE-negative individuals; this difference was significant (*t* = 8.38, d.f. = 2034.3, *P* < 0.001).The mean age of participants who completed a CAPE-P15 was 39.8 years (s.d. = 15.34) and it was 39.2 years (s.d. = 15.25) for participants who did not complete a CAPE-P15. There was no significant difference in age between those who did and did not complete a CAPE-P15 (*t* = 1.8127, d.f. = 2351.1, *P* = 0.07).

### Gender

The percentage of women to men was higher in both the CAPE-positive and CAPE-negative groups (67.6 and 70.4% respectively); however, when CAPE-positive and CAPE-negative individuals were compared, this gender difference was not significant (χ^2^ = 1.82, d.f. = 1, *P* = 0.193). Although more women (68.9%) completed a CAPE-P15 than men (31%), this gender difference was not significant when comparing participants who completed and did not complete a CAPE-P15 (χ^2^ = 1.0206, d.f. = 1, *P* = 0.3124).

### Ethnicity

Table 1 reveals that most people in this sample were White. Neither the difference between CAPE-positive and CAPE-negative individuals, nor the difference between those who did or did not complete a CAPE-P15 was significant (χ^2^ = 10.1, d.f. = 6, *P* = 0.122; χ^2^ = 12.225, d.f. = 6, *P* = 0.06 respectively).

### Prevalence of psychotic experiences

Table 2 shows the total number of CAPE-P15 assessments completed by patients receiving treatment from IAPT services in each of the three participating sites. The prevalence of CAPE-positive individuals, as defined by a score of 1.30 or above, ranged from 43 to 52%.

**Table 2:** Prevalence of psychotic experiences among patients across three services delivering the Improving Access to Psychological Therapies (IAPT) programme

	CPFT (*n* = 590), *n* (%)	NSFT (*n* = 1073), *n* (%)	SPFT (*n* = 379), *n* (%)	All sites (*n* = 2042), *n* (%)
CAPE-positive	253 (42.9)	557 (51.9)	172 (45.4)	982 (48.1)
CAPE-negative	337 (57.1)	516 (48.1)	207 (54.6)	1060 (51.9)

CPFT, Cambridgeshire and Peterborough NHS Foundation Trust; NSFT, Norfolk and Suffolk NHS Foundation Trust; SPFT, Sussex Partnership NHS Foundation Trust; CAPE-positive, scored ≥1.30 on the Community Assessment of Psychic Experiences (CAPE-P15); CAPE-negative, scored <1.30 on the CAPE-P15.

### Recovery rates

Table 3 compares the proportion of patients ending treatment in recovery between CAPE-positive and CAPE-negative individuals. Recovery rates for CAPE-negative individuals at all three sites fell within the nationally reported range. The percentage of CAPE-positive individuals who had reached recovery by the end of treatment was lower (26–50%) than the percentage of CAPE-negative individuals (55–69%).

**Table 3:** Recovery rates for patients with and without psychotic experiences across three services delivering the Improving Access to Psychological Therapies (IAPT) programme

Site	CAPE-positive, *n* (%)	CAPE-negative, *n* (%)
CPFT (*n* = 590)	253 (42.9)	337 (57.1)
NSFT (*n* = 1073)	557 (51.9)	516 (48.1)
SPFT (*n* = 379)	172 (45.4)	207 (54.6)
All sites (*n* = 2042)	982 (48.1)	1060 (51.6)

CAPE-positive, scored ≥1.30 on the Community Assessment of Psychic Experiences (CAPE-P15); CAPE-negative, scored <1.30 on the CAPE-P15; CPFT, Cambridgeshire and Peterborough NHS Foundation Trust; NSFT, Norfolk and Suffolk NHS Foundation Trust; SPFT, Sussex Partnership NHS Foundation Trust.

### Improvement trajectories

**Fig. 1:**
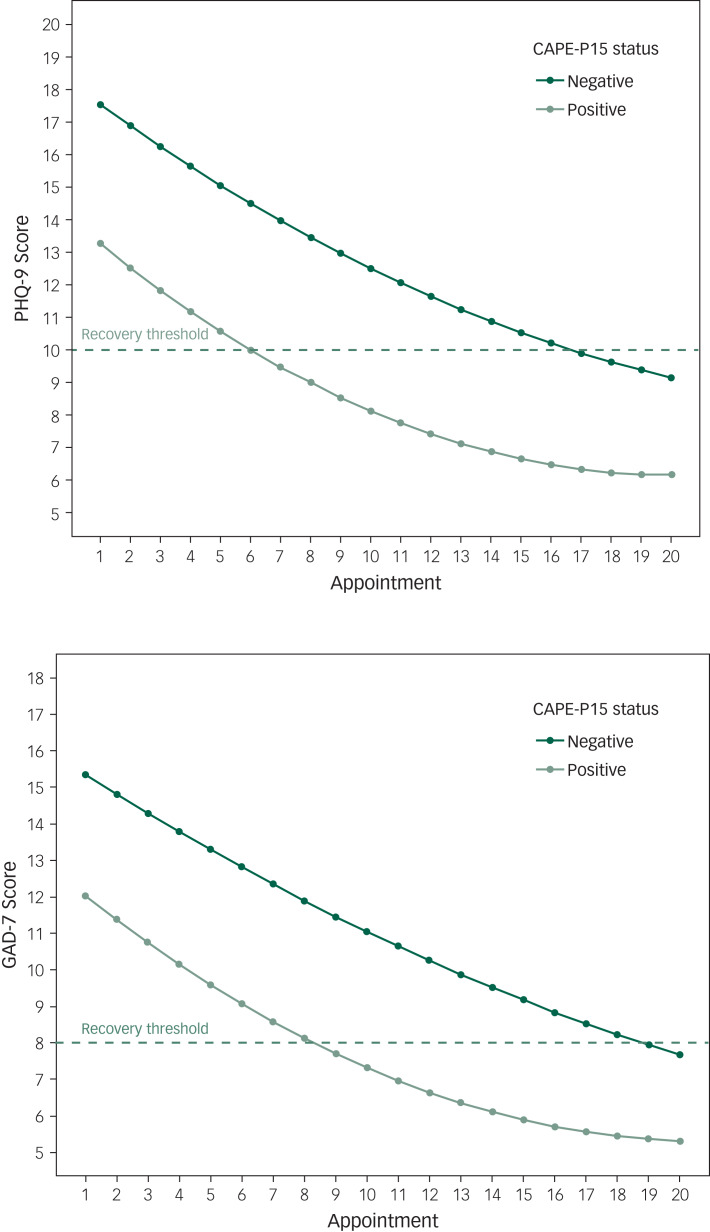
Trajectories of improvement on the Patient Health Questionnaire (PHQ-9) and Generalised Anxiety Disorder questionnaire (GAD-7) for participants with and without psychotic experiences. Positive, scored ≥1.30 on the Community Assessment of Psychic Experiences (CAPE-P15); negative, scored <1.30 on the CAPE-P15.

Figure 1 shows the mean improvement trajectories for CAPE-positive and CAPE-negative individuals for both the PHQ-9 and GAD-7. The initial CMD severity for CAPE-positive individuals was higher than for CAPE-negative individuals on both measures. CAPE-positive individuals began therapy with average scores of 17.5 and 15.5 on the PHQ-9 and GAD-7 respectively, compared with CAPE-negative individuals, who entered treatment with scores of approximately 13 and 12.

Although the improvement rates (trajectory slopes) were similar for both groups, beginning treatment with more severe depression and anxiety meant that CAPE-positive individuals required many more sessions to reach recovery thresholds (on average) compared with CAPE-negative individuals. For both the PHQ-9 and GAD-7, CAPE-positive individuals required on average 10 more sessions to reach recovery than CAPE-negative individuals on both measures: 16 sessions versus 6 on the PHQ-9 and 18 versus 8 on the GAD-7.

## Discussion

Our study confirmed that a significant proportion of people using primary mental healthcare services have comorbid CMD and psychotic experiences, resulting in poorer treatment outcomes by the end of therapy. Using a lower threshold score of 1.30 on the CAPE-P15, this study found that approximately half of all patients receiving IAPT treatment reported psychotic experiences. Only 36% of this group reached recovery by the end of treatment, compared with 64% without psychotic experiences. Although the overall reduction in CAPE-P15 score was minimal, it was significant and increased the identification of CAPE-positive individuals by 18% compared with the previously used cut-off of 1.47.^14^

This study also confirmed our hypothesis that patients with psychotic experiences would demonstrate more clinically severe CMD than those without psychotic experiences. Analysis of recovery trajectories showed that those with psychotic experiences entered treatment with higher initial scores on measures of depression (PHQ-9) and anxiety (GAD-7) and required many more treatment sessions, on average, to move from caseness to non-caseness. This may appear inevitable, given that individuals with more severe CMD require a larger reduction in scores to reach recovery than those beginning treatment with scores pertaining to mild or moderate CMD. IAPT services, however, are time limited and do not have the capacity to offer unlimited therapy sessions, especially when you consider that the more severe cases comprised half of the sample in this study. Nationally, patients finally receive an average of seven sessions of treatment.^27^

Attention should be directed at steepening the slope of the improvement trajectory rather than increasing the number of sessions. Here, we have shown that severity in CMD can be indexed by the presence of psychotic experiences, but IAPT services do not currently measure or provide interventions for those with these symptoms in addition to depression and anxiety disorders. Existing interventions that treat co-occurring CMD and psychotic experiences are not currently included in IAPT protocols and focus less on severe CMD and more on preventing transition to first-episode psychosis, with a resultant decrease in depression and anxiety.^28^ We suggest that a new conceptualisation of the co-occurrence of CMD and psychotic experiences is necessary: that psychotic experiences may dictate a continuum of CMD rather than just a continuum of psychosis. This is especially true for the population accessing primary mental healthcare services specifically for the treatment of CMD and whose recovery is assessed on the basis of a reduction of depression and anxiety scores. Treatment therefore should be evidence based and focus on these disorders. Moreover, these patients likely have treatment goals closely aligned to their CMD. However, focusing on mood disturbance alone oversimplifies the clinical reality and leaves psychotic experiences untreated, potentially exacerbating depression and anxiety.

This study represents an important work stream of a wider, innovative UK National Institute for Health Research (NIHR) Programme Grant for Applied Research (TYPPEX), where we propose that existing treatment protocols delivered by IAPT services could be tailored for people with more severe CMD, indexed by the presence of distressing psychotic experiences. Existing evidence-based treatments for both CMD and at-risk mental states for psychosis could be restructured to increase the potential for recovery among these individuals. Identifying psychotic experiences, understanding how they contribute to clinical outcomes and reconfiguring familiar CBT-based techniques into existing IAPT treatment protocols would provide a therapy tailored to individual needs.

### Limitations

This study provides practical evidence of the potential flexibility of CAPE-P15 threshold scores to identify more people with less disabling, but nonetheless debilitating psychotic experiences and facilitate access to more specific, tailored interventions in IAPT services. Nevertheless, the interpretation of the findings must consider the following limitations.

First, as CAPE-P15 completion was not mandated across our sites (unlike the completion of the PHQ-9 and GAD-7), CAPE-P15 data were obtained for only a small proportion of the overall case-load. In addition, selection bias was eliminated at only one site (NSFT), where requests for completion of clinical measures were automatically distributed to new patients via email. Conversely, therapists in the other two sites could exercise discretion when asking patients to complete a CAPE-P15 questionnaire. This could, hypothetically, have inflated the prevalence of psychotic experiences in these sites owing to the opportunity for therapists to select patients with more complex illness to facilitate an understanding of their presentation. However, reassurance that this was not the case is found in the following data: (a) the highest prevalence of psychotic experiences was found in the site that automated CAPE-P15 collection (Table 3); (b) recovery outcomes were similar across all sites; (c) sociodemographic variables did not differ between those who completed the CAPE-P15 and those who did not across all sites.

Second, increasing the CAPE-P15's sensitivity reduces its specificity. Lowering the threshold scores to 1.30 for frequency of psychotic experiences and associated distress not only increases the probability of identifying more people with CMD who have psychotic experiences, but also increases the risk of false positives. However, if the aim of identification is to deliver low-risk yet potentially beneficial treatments, compromising specificity to increase access to treatment may be valid.

### Implications

Growing evidence suggests that psychotic experiences may be a useful marker of severity in CMD. Consequently, the benefits of identifying higher-threshold severe psychotic experiences in an IAPT population^14^ may also apply to patients with milder psychotic experiences. Future research should investigate the tolerability and safety of such interventions and whether higher demands on service provision could be met without affecting routine clinical practice.

## Data Availability

The data that support the findings of this study are available from the corresponding author, J.P., upon reasonable request.
