# More sensitive identification of psychotic experiences in common mental disorder by primary mental healthcare services – effect on prevalence and recovery: casting the net wider – CORRIGENDUM

**DOI:** 10.1192/bjo.2020.151

**Published:** 2021-01-29

**Authors:** Clare Knight, Debra Russo, Jan Stochl, Peter B. Jones, Jesus Perez

**Keywords:** Anxiety, at-risk mental state, common mental disorder, depression, psychotic experiences

Soon after publication, the authors realised that Table 3 of this manuscript is not correct and must be changed. Unfortunately, during the review process a duplicate of Table 2, in a different format, replaced their original Table 3 and they overlooked this technical error. Thus, Table 3 must be replaced with the original one (below), which reflects the results as reported in both abstract and main text. They sincerely apologise for this error.

**Table 3.** Recovery rates for patients with and without psychotic experiences across three services delivering the Improving Access to Psychological Therapies (IAPT) programme.

Site	Status at discharge	CAPE-P15 positive	CAPE-P15 negative
CPFT	Not recovered	134 (57.8%)	91 (29.0%)
	Recovered	90 (38.8%)	215 (68.5%)
	Missing data	8 (3.5%)	8 (2.6%)
NSFT	Not recovered	203 (74.4%)	108 (45.0%)
	Recovered	70 (25.6%)	131 (54.6%)
	Missing data	0 (0%)	1 (0.42%)
SPFT	Not recovered	65 (45.8%)	46 (25.7%)
	Recovered	71 (50.0%)	124 (69.3%)
	Missing data	6 (4.2%)	9 (5.0%)
	Not recovered	402 (62.1%)	245 (33.4%)
All sites	Recovered	231 (35.7%)	470 (64.1%)
	Missing data	14 (2.2%)	18 (2.5%)

CAPE-positive, scored ≥1.30 on the Community Assessment of Psychic Experiences (CAPE-P15); CAPE-negative, scored <1.30 on the CAPE-P15; CPFT, Cambridgeshire and Peterborough NHS Foundation Trust; NSFT, Norfolk and Suffolk NHS Foundation Trust; SPFT, Sussex Partnership NHS Foundation Trust.
